# Recent Teaching on Their Surgical Diagnosis and Treatment

**Published:** 1920-05-15

**Authors:** 


### GASTRIC ULCERS.

### Recent Teaching on their Surgical Diagnosis and Treatment.

Present-day statistics have revealed the disquieting fact that many gastric ulcers are malignant, and even if innocent at the commencement of disease tend, in a proportion of cases, to become malignant at their termination. Students will often ask their teachers this most important question: "When does a chronic but otherwise innocent gastric ulcer become malignant," and the answer invariably given is that "we do not know." There are, it is true, a plethora of scientific observations which help to confirm a diagnosis, but they cannot be regarded as other than "aids to diagnosis." This is the crux of the new teaching, for previously much too great an amount of time was spent in performing "test meals," and in skiagraphing the gastro-intestinal tract subsequent to a "Bismuth Meal."

### The Fallacy of Gastric Analysis.

For some years chemical pathologists have described a definite decrease in the amount of free hydrochloric acid in the stomach afflicted by carcinoma: but more recently an actual increase of the free acid has been discovered in cases of early carcinoma. Thus the value of such a test has fallen from its former "high estate" and is no longer treated very seriously by gastric surgeons. Moreover, such tests take up a good deal of time and are spoken of by Mayo as "this scientific and deadly delay."

A very strong condemnation, but quite justified; and that brings us to the other great item in modern teaching on this subject, "the urgency for laparotomy and possible operation in *all* cases of suspected gastric ulcer." As great an authority as Sir William Hale-White has made the following impressive dictum: "Indigestion or dyspepsia in a subject of middle age, which does not improve after a short course of medical treatment, should be explored by abdominal section."

"Without operation cancer of the stomach is absolutely hopeless," said Mr. R. P. Rowlands in a clinical lecture delivered at Guy's Hospital last month. Spontaneous cure is not known, and medicine is of no use except to alleviate the symptoms of inoperable disease. Surely surgery can do better than this if given a fair chance; but too often the surgeon is not asked to see the patient until the diagnosis has become obvious and the prognosis is hopeless.

### Early Diagnosis.

Cancer of the stomach, in its early stages, does not give rise to symptoms which can be said to be typical of the disease. Rapid and progressive wasting, anæmia, pain, anorexia, nausea, and vomiting are suggestive. These, and especially if accompanied by a palpable tumour or obstructive signs, will indicate the necessity for an early operation. Mayo and Rowlands advocate a very simple measure for the determination of gastric delay or obstruction, which in their opinion is of greater value than either the "test meal" or an $x$-ray examination. Some half-cooked rice and a few raisins are given with some soup in the evening, and if on washing out the stomach in the morning any food remnants are found there is clearly obstruction; and, even though this may be caused by a simple ulcer, it strongly indicates an operation, both for diagnosis and treatment.

### Results of Partial Gastrectomy.

In the past the most common operation for gastric ulcer, whether innocent or malignant, was gastro-jejunostomy, by which the posterior surface of the stomach was united by anastomosis with the proximal loop of the jejunum. Such a measure relieves pyloric obstruction, but in the case of cancer is only palliative, unless some attempt is also made to eradicate the malignant disease itself. The American surgeon Mayo was the first to secure valuable results from an operation which he termed "Partial Gastrectomy." In this country the performance of gastrectomy was delayed for some years, because the immediate mortality was supposed to be very high, and the prospect of permanent cure or prolonged relief was believed to be poor.

In regard to this question of immediate mortality, Mr. Rowlands says: "The technique of partial gastrectomy has been so much improved in recent years that the mortality, in good hands, should not be more than 10 per cent., but resection for definite malignant disease is not likely to show such good results until the patients are sent for operation much earlier than they are in this country at the present time." "In early cases the operation is easy and comparatively safe, whereas in late cases it may be very difficult, prolonged, and dangerous. Therefore the careful selection of cases for operation is necessary." As regards the ultimate results, Mayo states that the patient with carcinoma of the stomach, which is sufficiently localised to be removed, has at least a 25 per cent. chance of a five years' cure.

### Indications for Operation.

At the present time less than a quarter of the cases come to the surgeon soon enough to allow resection to be performed; nearly two-thirds are unsuitable for any operation, but in nearly a quarter of the cases an exploration is necessary to decide whether anything can be done. The danger of an early operation is very small, although a simple exploration in late and inoperable cases carries with it a considerable risk, as shown by the experience of Kronlein and Mikulicz, who had a mortality of about 9 per cent. in such late explorations.

Clinical signs which indicate the growth to be inoperable are as follows: (1) Large size and fixation

**Gastric Ulcers**—(*continued*).

of the growth; (2) the discovery of dropped and grafted nodules of growth in the pelvis; (3) enlarged supra clavicular glands, especially common on the left side; (4) nodules of growth at the umbilicus, or under the skin of the abdomen; (5) ascites, indicating obstruction of the portal vein by growth; (6) nodular enlargement of the liver; and (7) an exhausted and cachetic state of the patient.

An accurate diagnosis of the early stages of malignant growth can only be obtained by an exploratory operation, and this procedure, if performed early enough, is associated with little risk to the life of the patient. Partial gastrectomy is the great hope for these frequent cases of malignant ulcer. Its technique is gradually improving, and if only cases could be diagnosed more early the present mortality from this most distressing disease might be reduced by one-half, or even more.

